# CT Angiography-Based Radiomics for Classification of Intracranial Aneurysm Rupture

**DOI:** 10.3389/fneur.2021.619864

**Published:** 2021-02-22

**Authors:** Osamah Alwalid, Xi Long, Mingfei Xie, Jiehua Yang, Chunyuan Cen, Huan Liu, Ping Han

**Affiliations:** ^1^Department of Radiology, Union Hospital, Tongji Medical College, Huazhong University of Science and Technology, Wuhan, China; ^2^Hubei Province Key Laboratory of Molecular Imaging, Wuhan, China; ^3^School of Electronic Information and Communications, Huazhong University of Science and Technology, Wuhan, China; ^4^GE Healthcare, Shanghai, China

**Keywords:** intracranial aneurysm, aneurysm rupture, subarachnoid hemorrhage, machine learning, radiomics

## Abstract

**Background:** Intracranial aneurysm rupture is a devastating medical event with a high morbidity and mortality rate. Thus, timely detection and management are critical. The present study aimed to identify the aneurysm radiomics features associated with rupture and to build and evaluate a radiomics classification model of aneurysm rupture.

**Methods:** Radiomics analysis was applied to CT angiography (CTA) images of 393 patients [152 (38.7%) with ruptured aneurysms]. Patients were divided at a ratio of 7:3 into retrospective training (*n* = 274) and prospective test (*n* = 119) cohorts. A total of 1,229 radiomics features were automatically calculated from each aneurysm. The feature number was systematically reduced, and the most important classifying features were selected. A logistic regression model was constructed using the selected features and evaluated on training and test cohorts. Radiomics score (Rad-score) was calculated for each patient and compared between ruptured and unruptured aneurysms.

**Results:** Nine radiomics features were selected from the CTA images and used to build the logistic regression model. The radiomics model has shown good performance in the classification of the aneurysm rupture on training and test cohorts [area under the receiver operating characteristic curve: 0.92 [95% confidence interval CI: 0.89–0.95] and 0.86 [95% CI: 0.80–0.93], respectively, *p* < 0.001]. Rad-score showed statistically significant differences between ruptured and unruptured aneurysms (median, 2.50 vs. −1.60 and 2.35 vs. −1.01 on training and test cohorts, respectively, *p* < 0.001).

**Conclusion:** The results indicated the potential of aneurysm radiomics features for automatic classification of aneurysm rupture on CTA images.

## Introduction

The incidence of intracranial aneurysm is ~3% in the adult population at a mean age of 50 years (1, 2). Aneurysms are responsible for about 80–90% of subarachnoid hemorrhages (SAH), with a resultant mortality rate of 23–51%, and a permanent disability risk of 10–20% (3, 4). Proper prevention is essential to reducing the risk of aneurysm rupture in the majority of cases, and timely management in case of rupture is critical for reducing the complications and preventing re-bleeding (2, 5, 6).

CT angiography is the first-line imaging examination for assessing cerebral aneurysms, with a reported sensitivity and specificity of 98 and 100%, respectively (7, 8). CT angiography is a fast and cost-effective diagnostic technique with a wide availability and high spatial resolution. Compared with digital subtraction angiography, which is the gold standard for diagnosing intracranial aneurysms, CT angiography is non-invasive and more widely available (6, 9).

Radiomics is a newly emerging technology that automatically extracts features from medical imaging to quantify the corresponding phenotypic characteristics (10). There is a trend of increasing interest in radiomics features as non-invasive imaging tools for estimation of pathological or histological features, distinction of benign and malignant entities, prediction of prognosis or treatment response, and inference to the genetic expression (10–13). These imaging biomarkers possess a potential to be more cost effective and provide a more individualized medical care (11, 14, 15).

To our knowledge, no reported studies on the establishment of a radiomics diagnostic model of intracranial aneurysm rupture exist. Radiomics may enhance our understanding of the value and clinical utility of the voxel-level imaging phenotypic features of intracranial aneurysms. Automatic processes proved effective to triage radiology workflow and to reduce the time to diagnosis in acute neurological events (16). In this regard, a potential role of radiomics is to automate the classification of aneurysm rupture status. In our previous work, we have developed a deep learning-based algorithm for automatic detection of intracranial aneurysms (17). Herein, we continue to build a radiomics signature of ruptured aneurysms that may be integrated with the computer-assisted detection system for a comprehensive automated aneurysm detection and rupture classification.

Therefore, this study aimed to identify the aneurysm radiomics features associated with rupture and to build and evaluate a classification model on CT angiography, which may provide a basis for automated diagnosis of aneurysm rupture.

## Materials and Methods

Ethical approval for this retrospective study was obtained from the institutional review board, and informed consent was waived.

### Study Population

The inclusion criteria for this study were (1) adult patient over 18 years old and (2) a diagnosis of intracranial aneurysm on CT angiography regardless of the rupture status of the aneurysm. Exclusion criteria included (1) multiple aneurysms; (2) multiple scans (only one time scan, often the most recent scan was selected per case); (2) non-saccular (fusiform or dissecting), traumatic, infectious, and previously treated aneurysms; (3) CTA images with severe motion artifact; (4) cases with unavailable clinical record; and (5) cases with unextractable radiomics features for the segmented lesion due to too few dimensions according to the feature extraction platform.

**Figure 1:**
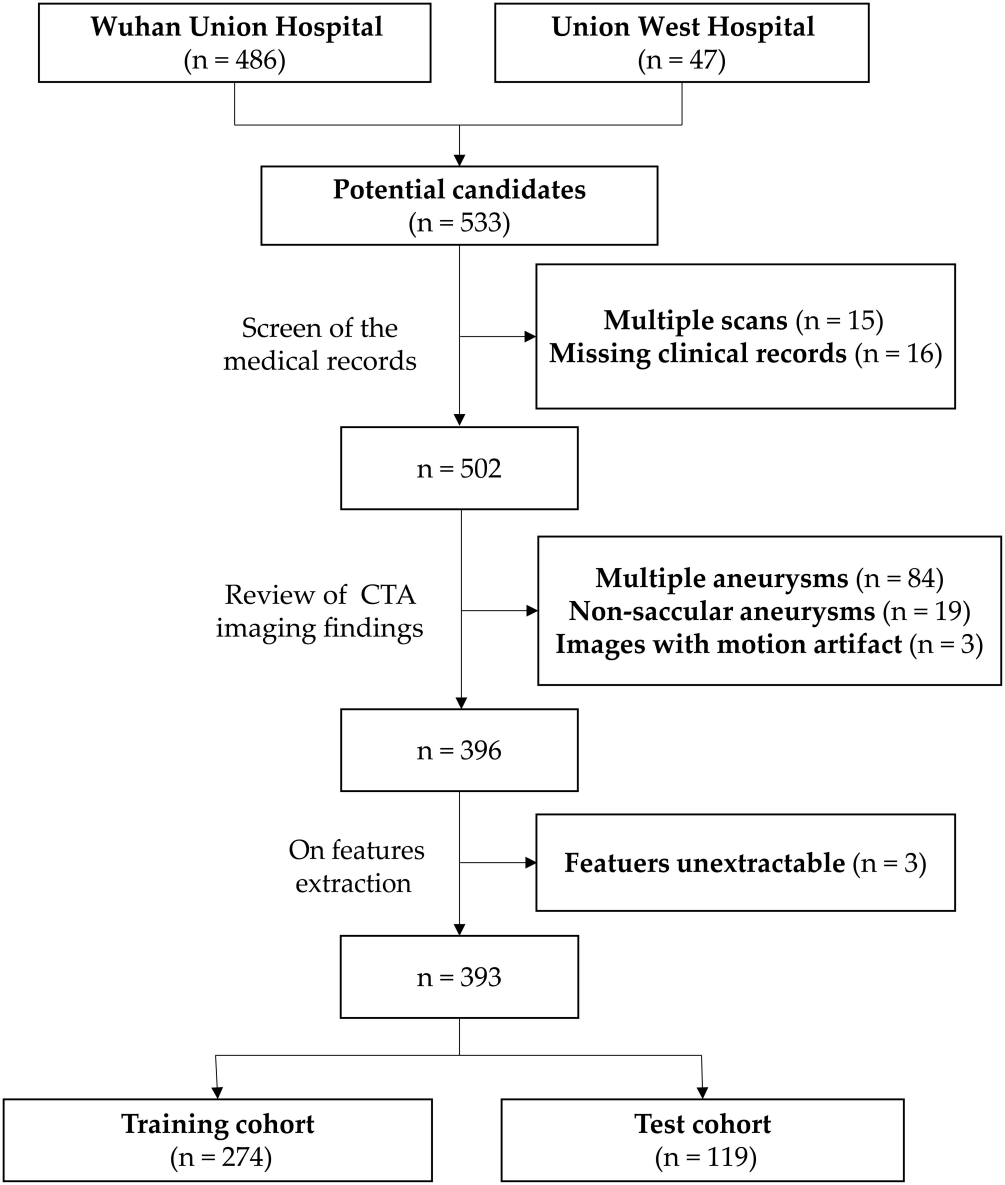
Flowchart of the patients' inclusion and exclusion process.

**Figure 2:**
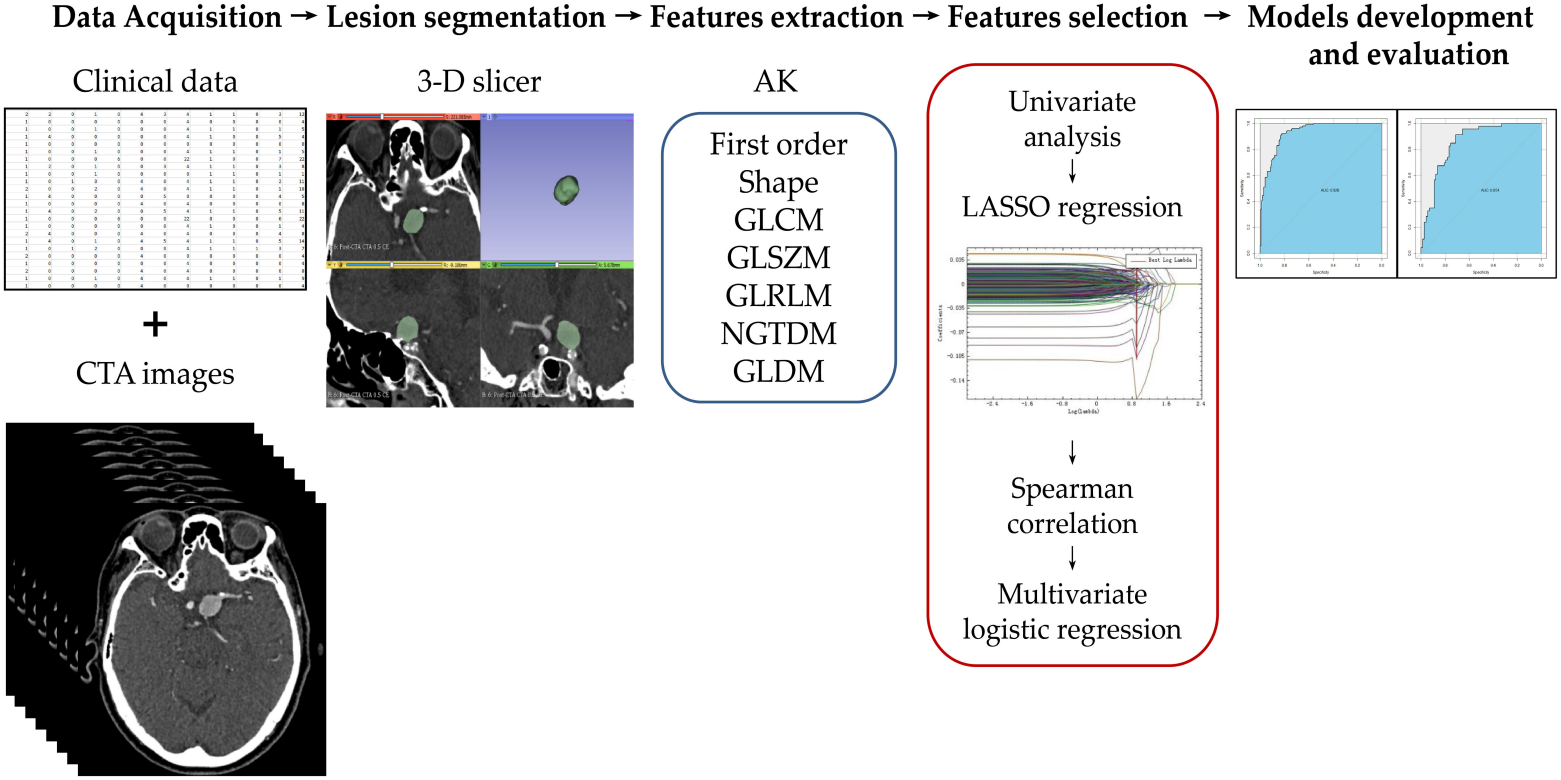
Illustration of the study workflow. Clinical and imaging data of the potential candidates were collected and assessed for enrollment eligibility. The aneurysms were segmented by two radiologists using 3D slicer software. Seven groups of radiomics features were extracted using Artificial Intelligence Kit (AK). The feature number was reduced using a step-wise process. The selected radiomics features were used to build the radiomics model. The model was evaluated using the area under receiver operating characteristic curve on training and test cohorts.

A flowchart of the patients' inclusion and exclusion process is shown in Figure 1, and the study workflow is summarized in Figure 2.

### Clinical and Imaging Data

CT angiography (CTA) imaging data of the patients with intracranial aneurysm diagnosed between May 2016 and April 2019 were collected from Wuhan Union Hospital and Union West Hospital. Patients were divided into two groups based on aneurysm rupture status as follows: the ruptured aneurysm group included patients with spontaneous subarachnoid hemorrhage documented by conventional brain CT with nearby aneurysm identified on CT angiography and confirmed by digital subtraction angiography, without any other potential pre-disposing factor (trauma, dissection, or local or systemic infection), and the unruptured aneurysm group included patients with intracranial aneurysm but no subarachnoid hemorrhage or related clinical symptoms.

Patients' clinical data and CTA imaging findings were collected. Clinical data included sex, age, history of hypertension, smoking, or previous SAH, and subsequent surgical treatment of the aneurysm. Imaging findings included number, size, shape [regular (smooth with no lobulation or daughter sac) or irregular], and location of aneurysms [internal carotid artery; middle cerebral artery; anterior circulation (anterior cerebral artery, anterior communicating artery and posterior communicating artery); and posterior circulation (vertebral artery, basilar artery, and posterior cerebral artery)], as well as the presence or absence of subarachnoid hemorrhage. PHASES (Population, Hypertension history, Age of patient, Size of aneurysm, earlier SAH from another aneurysm, and site of aneurysm) score was calculated in accordance with previous reports by summing up the scores assigned to each variable (1, 18) as follows: population [0, North American, Chinese, or European (other than Finnish); 3, Japanese; 5, Finnish], history of hypertension (0, no; 1, yes), age (0, < 70 years; 1, ≥70 years), aneurysm size (0, <7.0 mm; 3, 7.0–9.9 mm; 6, 10.0–19.9 mm; 10, ≥20.0 mm), history of SAH (0, no; 1, yes), and aneurysm location (0, internal carotid artery; 2, middle cerebral artery; 4, anterior cerebral arteries, posterior communicating artery, and posterior circulation).

### Imaging Techniques

The imaging protocol included standard CT angiography of the head or head and neck acquired on one of four imaging scanners including Discovery CT750 HD (GE Healthcare, Chicago, IL, USA; *n* = 161), SOMATOM Definition AS + (Siemens Healthineers, Erlangen, Germany; *n* = 111), and Aquilion ONE (Toshiba, Tokyo, Japan; *n* = 85) from Wuhan Union Hospital, and Ingenuity CT scanner (Philips Healthcare, Best, The Netherlands; *n* = 36) from Union West Hospital. The imaging protocols are summarized in the Supplementary Materials.

### Image Segmentation, Pre-processing, and Feature Extraction

Two general radiologists with 5 and 7 years of experience in head CTA independently manually segmented the region of interest (ROI) around the intracranial aneurysm slice by slice on three orthogonal views (axial, sagittal, and coronal) using 3D slicer 4.10.1 (https://www.slicer.org/).

Before feature extraction, image pre-processing with registration and resampling to a uniform pixel dimension of 1.0 × 1.0 × 1.0 mm^3^ with linear and nearest-neighbor interpolation for CTA and segmentation images, respectively, was performed using AK software (Artificial Intelligence Kit, Version V3.2.2.R, GE Healthcare). Seven feature groups were extracted, including first-order statistics, shape, gray-level co-occurrence matrix (GLCM), gray-level size-zone matrix (GLSZM), gray-level run-length matrix (GLRLM), neighborhood gray-tone difference matrix (NGTDM), and neighboring gray-level dependence matrix (GLDM). To enhance intricate patterns in the data invisible to the human eye (12), advanced filters including Laplacian of Gaussian (LoG; sigma, 2.0 and 3.0 mm), wavelet decompositions with all possible combinations of high- (H) or low- (L) pass filter in each of the three dimensions (HHH, HHL, HLH, LHH, LLL, LLH, LHL, HLL), and local binary pattern (LBP; level, 2; radius, 1.00) were applied. A total of 1,229 radiomics features were extracted from each aneurysm. Definitions and calculations of the radiomics features used in this study are available in the PyRadiomics documentation (http://PyRadiomics.readthedocs.io/en/latest/) (19).

The reproducibility of the features was assessed by means of intra- and inter-reader agreement for radiomics features using 58 randomly chosen cases incorporating a balanced number of images from each of the four scanners. To evaluate the intra-reader agreement, reader 1 performed the region-of-interest (ROI) segmentation twice with 1-month interval time. Meanwhile, reader 2 independently segmented the same set of images once to assess the inter-reader agreement with the radiomics features extracted from the first ROI segmented by reader 1. Intraclass correlation coefficient (ICC) was performed to assess the intra- and inter-reader agreement. An ICC cutoff of >0.8 was selected for accepted reproducibility level of the radiomics features.

### Dimensionality Reduction and Radiomics Feature Selection

Feature extraction and dimensionality reduction were performed using AK software. A stepwise process was carried out to select the features in the training cohort as shown in the study workflow (Figure 2). First, all features suggested by excellent ICC (>0.8) were assessed by one-way ANOVA or Mann–Whitney *U*-test to select the significant classifying features. Features that were not significantly different between ruptured and unruptured aneurysms were removed. Then, least absolute shrinkage and selection operator (LASSO) regression analysis was adopted for dimensionality reduction by performing variable selection and regularization using 10-fold cross-validation. The remaining features were assessed by the Spearman correlation test for severe linear dependence. Features with >0.90 correlation coefficient were excluded. Finally, multivariate logistic regression using the backward likelihood ratio elimination method was used to determine the most important and independent discriminating features. Radiomics score (Rad-score) for each patient was calculated by linear combination of the selected features weighted by their corresponding logistic regression coefficient.

### Model Development and Evaluation

Machine learning using logistic regression was used to build the radiomics model using the selected radiomics features. The radiomics model was assessed by calculating the area under the receiver operating characteristic curve (AUC) on both the training and test cohorts. Other performance metrics, including sensitivity, specificity, positive predictive value (PPV), negative predictive value (NPV), and accuracy were also calculated.

### Statistical Analysis

Statistical analyses were performed using SPSS (Version 25, IBM) and R (Version 4.0.2, https://www.r-project.org/). The R packages used in the analyses included psych, pROC, and e1071. *Z*-score normalization of the data was performed as a pre-processing step. The Shapiro–Wilk test was used to assess the normality of distribution. Univariate analysis was performed for comparing the clinical factors of ruptured and unruptured aneurysms by using the chi-square test or Fisher exact test for categorical variables, and Student *t*-test or Mann–Whitney *U*-test for continuous variables, where appropriate. Significant factors were assessed by multivariate logistic regression analysis. Receiver operating characteristic (ROC) curves were generated to assess the performance of the radiomics model on the training and test cohorts. Hosmer–Lemeshow test was performed to evaluate the goodness of fit of the radiomics model. The significance level was set at *p* = 0.05 for the basic statistical analyses and *p* = 0.01 for the selection of radiomics features.

## Results

### Patients and Aneurysm Characteristics

A total of 393 patients [234 (59.5%) females] with 393 intracranial aneurysms were included. There were 152 (38.6%) patients with ruptured aneurysms and evident subarachnoid hemorrhage. The demographic, clinical, and imaging characteristics of the study population and data division are summarized in Table 1. Given the small number of cases from Union West Hospital (36/393, 9.2%) hindering solo use for validation, the entire data from two hospitals were mixed and randomly divided into training and testing cohorts at a ratio of 7:3 (274 cases for retrospective training and 119 cases for prospective testing). As evident from Table 1, no significant differences were present between the training and test cohorts in patients' age, sex, patient category (in-patients vs. out-patients), number of cases imaged by each scanner, history of hypertension, smoking, or previous SAH, aneurysm size, location, and rupture status and the subsequent surgical treatment of the aneurysms (all *p* > 0.05).

**Table 1:** Study population characteristics and data division.

**Variable**	**Total** ***n*** **=** **393**		***P*-value**
	**Training cohort**	**Testing cohort**	
No. of patients	274 (70%)	119 (30%)	NA
Age in years, median (IQR)	55 (49–63)	57 (50–66)	0.20
Sex
Male	113 (41.2%)	46 (38.7%)	0.66
Female	161 (58.8%)	73 (61.3%)	
Patient category
Out-patient	148 (54.0%)	59 (49.6%)	0.44
In-patient	126 (46.0%)	60 (50.4%)	
Imaging scanner
TOSHIBA	57 (20.8%)	28 (23.5%)	
SIEMENS	82 (29.9%)	29 (24.4%)	0.62
GE medical systems	112 (40.9%)	49 (41.2%)	
Philips	23 (8.4%)	13 (10.9%)	
History of hypertension	69/133 (51.9%)	37/65 (56.9%)	0.55
History of smoking	27/132 (20.5%)	17/61 (27.9%)	0.27
History of previous SAH	11/160 (6.9%)	5/70 (7.1%)	1.00
Maximal aneurysm size in mm,	4.3 (3.1–6.2)	4.7 (3.5–6.6)	0.38
median (IQR)			
Aneurysm location
ICA	154 (56.2%)	67 (56.3%)	
MCA	44 (16.1%)	13 (10.9%)	0.50
Anterior circulation	64 (23.4%)	31 (26.1%)	
Posterior circulation	12 (4.4%)	8 (6.7%)	
Ruptured aneurysms	106 (38.7%)	46 (38.7%)	1.00
Subsequent surgical treatment	77/194 (39.7%)	35/91 (38.5%)	0.90

*Data are number of patients and percentages unless otherwise specified. ICA, internal carotid artery; IQR, interquartile range; MCA, middle cerebral artery; NA, not applicable; SAH, subarachnoid hemorrhage; SD, standard deviation*.

Clinical risk factors of aneurysm rupture in the study population are summarized in Table 2. Univariate analysis revealed five potential risk factors for aneurysm rupture, including age, aneurysm size, location, and shape, as well as PHASES score. Multivariate analysis showed the age, aneurysm size, location, and shape to be independent clinical risk factors for intracranial aneurysm rupture.

**Table 2:** Clinical risk factors for aneurysm rupture in the study population.

**Factor**	**Ruptured (*n* = 152)**	**Unruptured (*n* = 241)**	**Univariate analysis (*p*)**	**Multivariate analysis (*p*)**
Age, years (mean ± SD)	55 ± 9	57 ± 11	**0.005**	**0.02**
Sex			0.07	NA
Male	70 (46.1%)	89 (36.9%)		
Female	82 (53.9%)	152 (63.1%)		
Hypertension	36/63 (57.1%)	70/135 (51.9%)	0.49	NA
Smoking	14/60 (23.3%)	30/133 (22.6%)	0.91	NA
Previous SAH	4/61 (6.6%)	12/169 (7.1%)	0.89	NA
Aneurysm size, mm (mean ± SD)	6.1 ± 2.8	4.6 ± 2.6	** <0.001**	**0.03**
Aneurysm location			** <0.001**	** <0.001**
ICA	46 (30.3%)	175 (72.6%)		
MCA	30 (19.7%)	27 (11.2%)		
Anterior circulation	65 (42.8%)	30 (12.4%)		
Posterior circulation	11 (7.2%)	9 (3.7%)		
Aneurysm shape			** <0.001**	** <0.001**
Regular	61 (40.1%)	180 (74.7%)		
Irregular	91 (59.9%)	61 (25.3%)		
PHASES score (mean ± SD)	4.2 ± 2.5	2.1 ± 2.2	** <0.001**	0.31

*Data are number of patients and percentages unless otherwise specified. Significant p-values are highlighted in bold. Anterior circulation includes anterior cerebral artery, anterior communicating artery, and posterior communicating artery. Posterior circulation includes vertebral artery, basilar artery, and posterior cerebral artery. PHASES, Population, Hypertension history, Age of patient, Size of aneurysm, Earlier SAH from another aneurysm, and Site of aneurysm; NA, not applicable; SAH, subarachnoid hemorrhage; SD, standard deviation*.

### Inter-reader and Intra-reader Agreement

A total of 1,229 features were included in the intra-class correlation test. Features with ICC of <0.8 were excluded. A total of 32 features (27 overlapping features) were excluded based on both intra- and inter-reader agreement. Eventually, 1,197 features were selected for further analyses. The overall intra-reader agreement of the 1,197 features was excellent (mean ICC = 0.979; range, 0.804–1.000). The overall inter-reader agreement of the selected 1,197 features was also excellent (mean ICC = 0.976; range, 0.801–1.000).

### Dimensionality Reduction and Feature Selection

Of the 1,197 features included in the analysis, 762 features have shown to be significantly different (*p* <0.05) between ruptured and unruptured aneurysms in the training cohort. Of these, 67 features were selected by LASSO regression analysis, with the best-tuned regularization parameter λ of 0.9 found by 10-fold cross-validation. The features were further reduced to 42 features by excluding those with Spearman correlation coefficient of >0.90. Finally, multivariate logistic regression analysis with the backward likelihood ratio elimination method revealed nine features as the most important independent classifiers (*p* < 0.01, Table 3). The nine features were used to build the radiomics model and calculate the radiomics score for each patient as follows:

**Table 3:** Candidate radiomics features according to multivariate logistic regression analysis.

Feature^*^	**Coefficient**	***P^#^***	***OR***	**95% CI for *OR***
Wavelet-HHL.firstorder.Entropy	1.861	**0.000**	6.433	3.338–12.398
LBP-3D-m1.firstorder.90Percentile	−1.293	**0.000**	0.274	0.142–0.530
LBP-3D-m2.firstorder.Skewness	−0.618	**0.006**	0.539	0.348–0.835
LoG-sigma-20mm-3D.GLCM.ID	1.671	**0.000**	5.318	2.609–10.837
LoG-sigma-20mm-3D.GLSZM.SmallAreaHighGrayLevelEmphasis	1.097	**0.005**	2.994	1.386–6.467
LoG-sigma-30mm-3D.GLCM.InverseVariance	0.716	**0.003**	2.046	1.279–3.274
Original.GLSZM. SizeZoneNonUniformityNormalized	−0.665	0.014	0.514	0.302–0.876
Wavelet-LHH.firstorder.RootMeanSquared	−1.066	**0.007**	0.345	0.159–0.746
Wavelet-LHL.firstorder.Median	−0.689	**0.006**	0.502	0.308–0.817
Wavelet-LLH.GLDM.SmallDependenceEmphasis	−1.207	**0.001**	0.299	0.152–0.591
LoG-sigma-20mm-3D.firstorder.Skewness	0.513	0.038	1.671	1.030–2.711
LoG-sigma-30mm-3D.firstorder.10Percentile	0.996	0.041	2.708	1.040–7.046

* *Feature calculation is based on PyRadiomics (19)*.

# *Radiomics features with p-value of < 0.01 (shown in bold) made up the eventual radiomics signature. OR, odds ratio; 95% CI, 95% confidence interval*.

Rad-score = (Wavelet-HHL.firstorder.Entropy × 1.7182) + (LBP-3D-m1.firstorder.90Percentile × −1.2051) + (LBP-3D-m2.firstorder.Skewness × −0.4051) + (LoG-sigma-20mm-3D.GLCM.ID × 1.8458) + (LoG-sigma-20mm-3D.GLSZM.SmallAreaHighGrayLevelEmphasis × 0.5863) + (LoG-sigma-30mm-3D.GLCM.InverseVariance × 0.7882) + (Wavelet-LHH.firstorder.RootMeanSquared × −1.0978) + (Wavelet-LHL.firstorder.Median × −0.6913) + (Wavelet-LLH.GLDM.SmallDependenceEmphasis × −1.2181)

The Rad-score showed statistically significant differences between ruptured and unruptured aneurysms (median Rad-score of ruptured aneurysms: 2.50, range: −0.97–7.81; median Rad-score of unruptured aneurysms: −1.60, range: −14.11–4.55; *p* < 0.001 in the training cohort and median Rad-score of ruptured aneurysms: 2.35, range: −1.02–7.41; median Rad-score of unruptured aneurysms: −1.01, range: −11.53–5.19; *p* < 0.001 in the test cohort). The selected Rad-score cutoff value of 1.00 yielded a sensitivity of 87 and 79% and a specificity of 84 and 80% on training and test cohorts, respectively.

### Radiomics Model Performance

**Figure 3:**
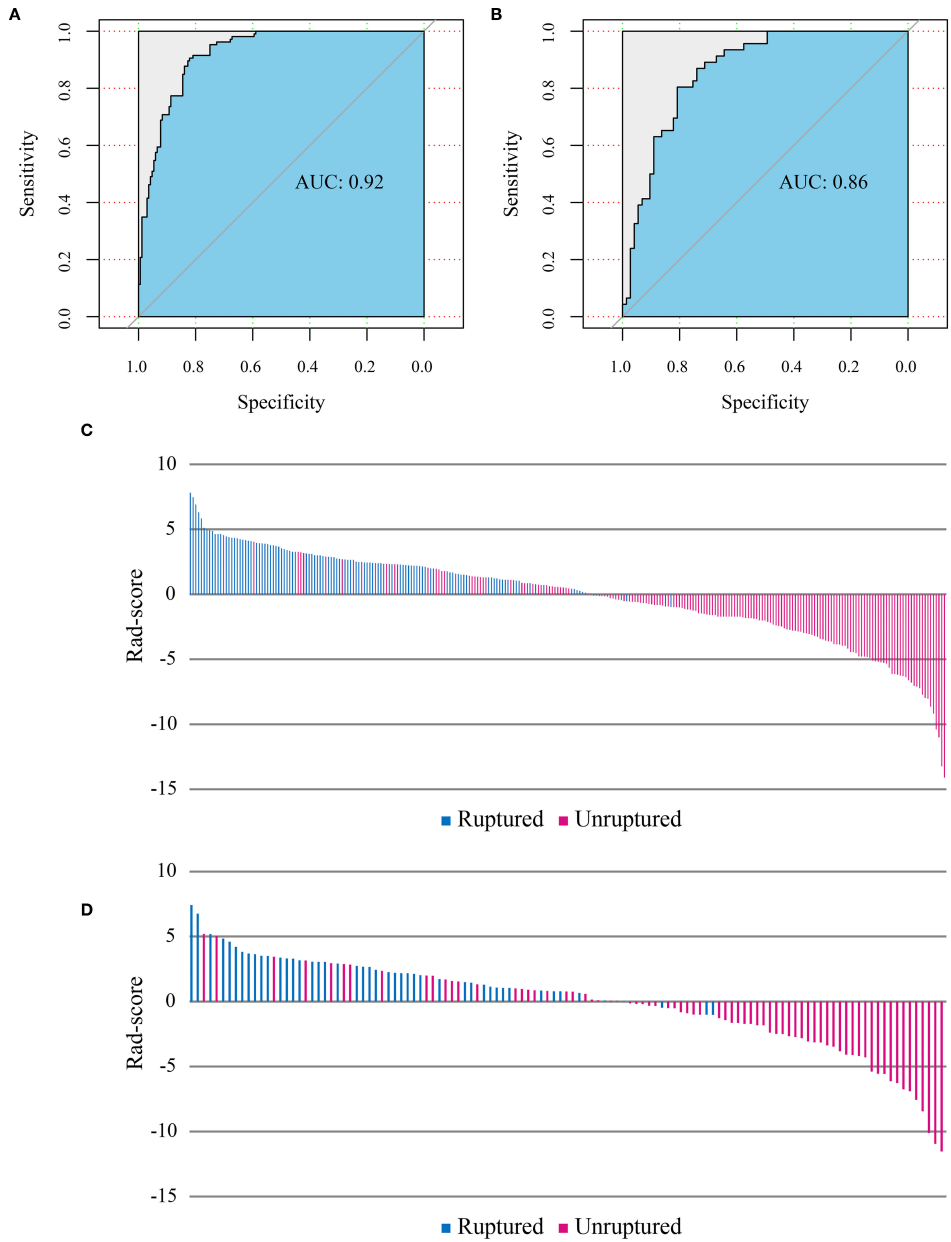
Receiver operating characteristic (ROC) curves of the radiomics model performance on training **(A)** and test cohorts **(B)**. Radiomics score (Rad-score) of each patient on training **(C)** and test cohorts **(D)** show the association of high Rad-score with risk of aneurysm rupture.

**Figure 4:**
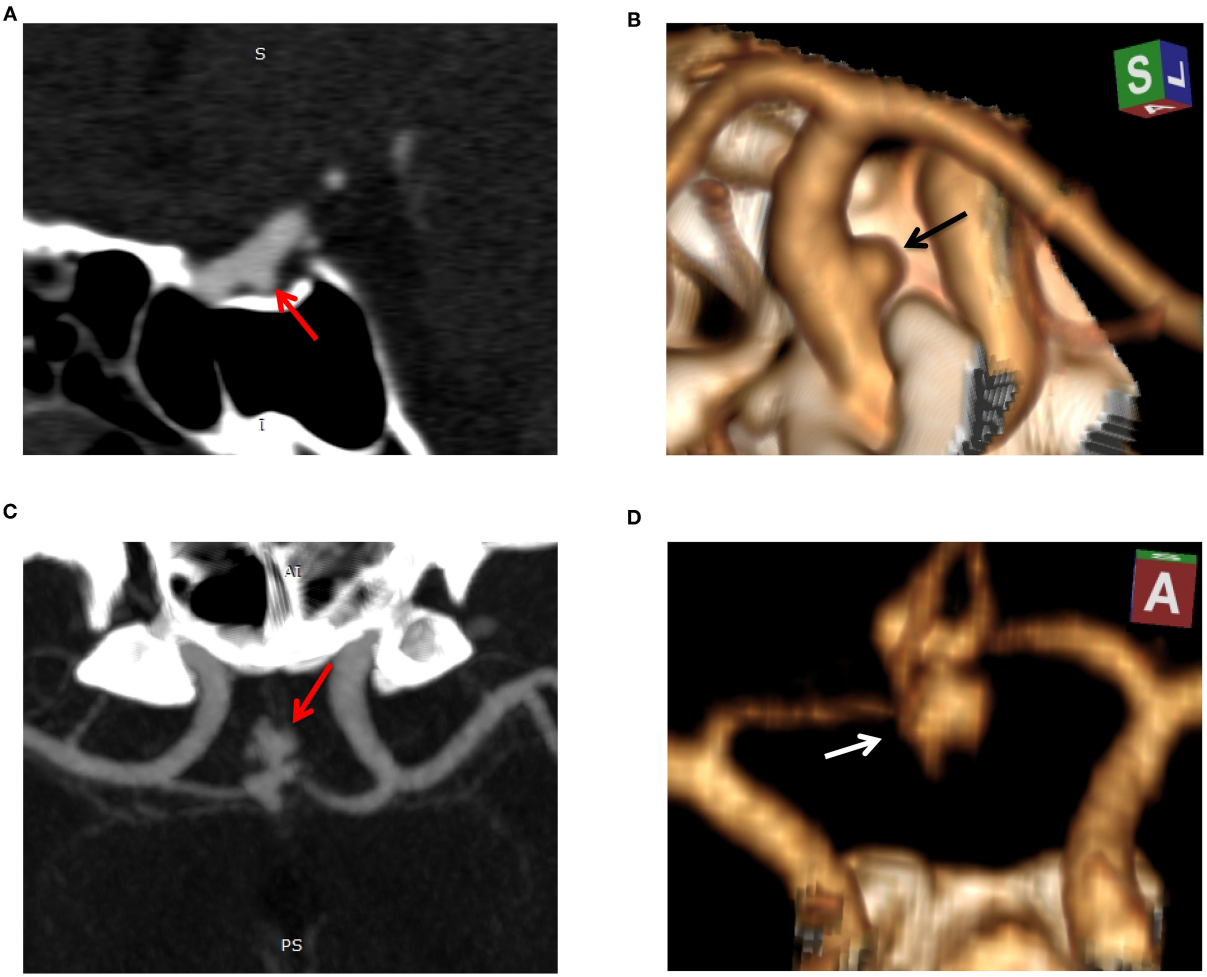
Example cases of unruptured and ruptured aneurysm features. Case 1: sagittal reconstructed maximum intensity projection (MIP, **A**) and 3D volume-rendered (3D-VR) CT angiography (CTA, **B**) images of a 52-year-old-male with unruptured aneurysm. The aneurysm was small (3.5 mm in maximal diameter), regular, and located on the internal carotid artery. Radiomics score was −6.1 indicating a low risk of rupture. Case 2: MIP **(C)** and 3D-VR CTA **(D)** images of a 50-year-old-male with ruptured aneurysm. The aneurysm measured 9.4 mm in maximal diameter, was irregular (lobulated with multiple daughter sacs), and located on the anterior communicating artery. Radiomics score was 3.5 indicating a high risk of rupture.

The radiomics model ROC curves and Rad-scores for each patient in the training and test cohorts are shown in Figure 3. The radiomics model has shown a good performance in classification of aneurysm rupture [AUC: 0.92 (95% CI: 0.89–0.95) and 0.86 (95% CI: 0.80–0.93) on training and test cohorts, respectively, *p* < 0.001]. On the training cohort, the accuracy, sensitivity, specificity, and positive and negative predictive values were 82, 77, 86, 77, and 86%, respectively. On the test cohort, the accuracy, sensitivity, specificity, and positive and negative predictive values were 76, 70, 81, 70, and 81%, respectively. The Hosmer–Lemeshow test showed a good fitness of the radiomics model on training and test cohorts (*P* = 0.78 and 0.83, respectively). Example cases from the study population are shown in Figure 4.

## Discussion

Intracranial aneurysm rupture is an acute neurological event with a high morbidity and fatality risk; therefore, an accurate and timely detection is critical. In this study, radiomics analysis was applied to CT angiography images of patients with ruptured and unruptured intracranial aneurysms, and a radiomics classification model was developed and evaluated. The radiomics model has shown a good performance in classification of aneurysm rupture on the training and test cohorts (AUCs of 0.92 and 0.86, respectively). The results indicate a great potential of radiomics signature as an automatic diagnostic marker of intracranial aneurysm rupture.

Clinically, several risk factors have been considered to be associated with intracranial aneurysm development, growth, and rupture (2, 5, 20, 21). Patients' age, sex, race, familial history, and history of hypertension, smoking, alcohol consumption, and previous stroke, as well as aneurysm size, multiplicity, location, and shape, are all reported risk factors, with variable results across studies mostly due to the variation in the study populations (2, 5, 22, 23). PHASES is a scoring system of six easily retrievable risk factors that was developed for prediction of aneurysm rupture risk (24). It had also shown a potential for prediction of aneurysm growth (1, 18). In our study population, younger age, larger aneurysm size, location on the middle cerebral artery, anterior or posterior circulation, and irregular shape were the independent clinical risk factors of aneurysm rupture, which is generally consistent with previous studies (2, 22, 25). It is noteworthy that controversy exists regarding the age as a risk factor for aneurysm rupture; however, it is an important contributing factor to the treatment decision (2).

Previous machine learning studies on intracranial aneurysm rupture status classification and rupture risk assessment have shown encouraging results. A recent study on morphologic and hemodynamic features of cerebral aneurysm on CTA revealed the projection ratio, irregular shape, and size ratio as important discriminators of ruptured aneurysms (26). Another study on clinical and imaging features has shown the location and size to have a strong association with aneurysm rupture (22). A study by Kim et al. (27) focusing on rupture status of small (<7 mm) aneurysms of anterior circulation on 3D digital subtraction angiography developed a CNN-based prediction system, which outperformed human readers. An earlier study on 60 aneurysms proposed a classification model of aneurysm rupture status using geometrical and wall shear stress parameters (28).

Radiomics has been successfully applied to intracranial aneurysm morphology analysis. Liu et al. (29) employed radiomics for identifying the morphological features associated with rupture in sidewall and bifurcation aneurysms on 3D digital subtraction angiography images. It was concluded that bifurcation configuration is an independent risk factor for aneurysm rupture regardless of the location. In our study, we have employed a large number of radiomics features with a systematic selection approach for building a classification model of aneurysm rupture on CT angiography. Radiomics features automatically extracted from PyRadiomics are calculated in a pixel-by-pixel manner and could closely reflect the morphologic features of the 3-dimensional object, such as an aneurysm (29). Radiomics calculation is a fast and automatic process once the structure of interest has been delineated; therefore, selected radiomics signature can be integrated with the automatic aneurysm detection systems, such as that developed by our team (17) or those reported in the literature (7, 30), for a comprehensive aneurysm detection and rupture classification. Automatic depiction of patients with ruptured aneurysms may also help prioritize the work list in radiology departments and facilitate the timely management of these patients.

Our study is limited by the retrospective enrollment, which carries a risk of selection bias. Second, although the radiomics calculation was automatic, the ROI segmentation was manual, which is prone to interoperator variability and hinders the clinical application of radiomics. Future utility of automatic segmentation might reduce the interoperator variability and improve the clinical feasibility of radiomics (31). Additionally, we had a heterogeneous data recruited from two institutions with CTA images acquired by four different scanners comprising various protocols. Large sample size and pre-processing techniques may partially control for the data heterogeneity (32). On the other hand, it was anticipated that training the machine learning models on heterogeneous data may improve the robustness and clinical feasibility (33). However, given that the reproducibility of radiomics studies is a common dilemma (10, 12, 34), therefore, our radiomics model likewise needs further exploration and validation on newly recruited external data.

In conclusion, our results demonstrated that a successful diagnostic classification of aneurysm rupture using radiomics features is achievable. As a non-invasive imaging tool, CTA-based radiomics analysis may provide a helpful practical method to automatically identify patients with ruptured intracranial aneurysm.

## Data Availability Statement

The datasets generated for this study are available on request to the corresponding author.

## Ethics Statement

The studies involving human participants were reviewed and approved by Institutional Review Board of Tongji Medical College, Huazhong University of Science and Technology. Written informed consent for participation was not required for this study in accordance with the national legislation and the institutional requirements.

## Author Contributions

OA, XL, and MX designed the study and collected the clinical data. XL and MX segmented the CTA images. JY, CC, and HL provided technical support and contributed to the data analysis. OA drafted the manuscript. PH edited the manuscript, supervised the entire study, and provided clinical expertise. All the authors discussed the results and read and approved the final version of the manuscript.

## Conflict of Interest

HL was employed by GE Healthcare Company. The remaining authors declare that the research was conducted in the absence of any commercial or financial relationships that could be construed as a potential conflict of interest.

## References

[1] Backes D Vergouwen MD Tiel Groenestege AT Bor ASE Velthuis BK Greving JP. PHASES score for prediction of intracranial aneurysm growth. Stroke. (2015) 46:1221–6. 10.1161/STROKEAHA.114.008198 25757900

[2] Thompson BG Brown RD Jr Amin-Hanjani S Broderick JP Cockroft KM Connolly ES Jr. Guidelines for the management of patients with unruptured intracranial aneurysms: a guideline for healthcare professionals from the American Heart Association/American Stroke Association. Stroke. (2015) 46:2368–400. 10.1161/STR.0000000000000070 26089327

[3] Van Gijn J Kerr RS Rinkel GJ. Subarachnoid haemorrhage. Lancet. (2007) 369:306–18. 10.1016/S0140-6736(07)60153-6 17258671

[4] Hop JW Rinkel GJ Algra A Van GJ. Case-fatality rates and functional outcome after subarachnoid hemorrhage: a systematic review. Stroke. (1997) 28:660. 10.1161/01.STR.28.3.660 9056628

[5] Steiner T Juvela S Unterberg A Jung C Forsting M Rinkel G. European Stroke Organization guidelines for the management of intracranial aneurysms and subarachnoid haemorrhage. Cerebrovasc Dis. (2013) 35:93–112. 10.1159/000346087 23406828

[6] Philipp LR McCracken DJ McCracken CE Halani SH Lovasik BP Salehani AA. Comparison between CTA and digital subtraction angiography in the diagnosis of ruptured aneurysms. Neurosurgery. (2017) 80:769–77. 10.1093/neuros/nyw113 28201559

[7] Park A Chute C Rajpurkar P Lou J Ball RL Shpanskaya K. Deep earning–Asasisted diagnosis of cerebral aneurysms using the HeadXNet model. JAMA Netw Open. (2019) 2:e195600. 10.1001/jamanetworkopen.2019.5600 31173130 PMC6563570

[8] Westerlaan HE van Dijk JM Jansen-van der Weide MC de Groot JC Groen RJ Mooij JJ. Intracranial aneurysms in patients with subarachnoid hemorrhage: CT angiography as a primary examination tool for diagnosis–systematic review and meta-analysis. Radiology. (2011) 258:134–45. 10.1148/radiol.10092373 20935079

[9] Yoon NK McNally S Taussky P Park MS. Imaging of cerebral aneurysms: a clinical perspective. Neurovasc Imaging. (2016) 2:6. 10.1186/s40809-016-0016-3

[10] Zwanenburg A Vallieres M Abdalah MA Aerts H Andrearczyk V Apte A. The image biomarker standardization initiative: standardized quantitative radiomics for high-throughput image-based phenotyping. Radiology. (2020) 295:328–38. 10.1148/radiol.2020191145 32154773 PMC7193906

[11] Wang Y Yu Y Han W Zhang YJ Jiang L Xue HD. CT Radiomics for distinction of human epidermal growth factor receptor 2 negative gastric cancer. Acad Radiol. (2020). 10.1016/j.acra.2020.02.018. [Epub ahead of print]. 32303442

[12] Papanikolaou N Matos C Koh DM. How to develop a meaningful radiomic signature for clinical use in oncologic patients. Cancer Imaging. (2020) 20:33. 10.1186/s40644-020-00311-4 32357923 PMC7195800

[13] Saini A Breen I Pershad Y Naidu S Knuttinen MG Alzubaidi S. Radiogenomics and radiomics in liver cancers. Diagnostics. (2018) 9:4. 10.3390/diagnostics9010004 30591628 PMC6468592

[14] Yu X Song W Guo D Liu H Zhang H He X. Preoperative prediction of extramural venous invasion in rectal cancer: comparison of the diagnostic efficacy of radiomics models and quantitative dynamic contrast-enhanced magnetic resonance imaging. Front Oncol. (2020) 10:459. 10.3389/fonc.2020.00459 32328461 PMC7160694

[15] Avanzo M Wei L Stancanello J Vallieres M Rao A Morin O. Machine and deep learning methods for radiomics. Med Phys. (2020) 47:e185–202. 10.1002/mp.13678 32418336 PMC8965689

[16] Titano JJ Badgeley M Schefflein J Pain M Su A Cai M. Automated deep-neural-network surveillance of cranial images for acute neurologic events. Nat Med. (2018) 24:1337–41. 10.1038/s41591-018-0147-y 30104767

[17] Yang J Xie M Hu C Alwalid O Xu Y Liu J. Deep learning for detecting cerebral aneurysms with CT angiography. Radiology. (2020) 298:155–63. 10.1148/radiol.2020192154 33141003

[18] Brinjikji W Pereira VM Khumtong R Kostensky A Tymianski M Krings T. PHASES and ELAPSS scores are associated with aneurysm growth: a study of 431 unruptured intracranial aneurysms. World Neurosurg. (2018) 114:e425–32. 10.1016/j.wneu.2018.03.003 29530704

[19] van Griethuysen JJM Fedorov A Parmar C Hosny A Aucoin N Narayan V. Computational radiomics system to decode the radiographic phenotype. Cancer Res. (2017) 77:e104–7. 10.1158/0008-5472.CAN-17-0339 29092951 PMC5672828

[20] Korja M Lehto H Juvela S. Lifelong rupture risk of intracranial aneurysms depends on risk factors: a prospective Finnish cohort study. Stroke. (2014) 45:1958–63. 10.1161/STROKEAHA.114.005318 24851875

[21] Jin D Song C Leng X Han P. A systematic review and meta-analysis of risk factors for unruptured intracranial aneurysm growth. Int J Surg. (2019) 69:68–76. 10.1016/j.ijsu.2019.07.023 31356963

[22] Silva MA Patel J Kavouridis V Gallerani T Beers A Chang K. Machine learning models can detect aneurysm rupture and identify clinical features associated with rupture. World Neurosurg. (2019) 131:e46–51. 10.1016/j.wneu.2019.06.231 31295616

[23] Ishibashi T Murayama Y Urashima M Saguchi T Ebara M Arakawa H. Unruptured intracranial aneurysms: incidence of rupture and risk factors. Stroke. (2009) 40:313–6. 10.1161/STROKEAHA.108.521674 18845802

[24] Greving JP Wermer MJ Brown Jr RD Morita A Juvela S Yonekura M. Development of the PHASES score for prediction of risk of rupture of intracranial aneurysms: a pooled analysis of six prospective cohort studies. Lancet Neurol. (2014) 13:59–66. 10.1016/S1474-4422(13)70263-1 24290159

[25] Wang GX Wen L Lei S Ran Q Yin JB Gong ZL. Wall enhancement ratio and partial wall enhancement on MRI associated with the rupture of intracranial aneurysms. J Neurointerventional Surg. (2018) 10:566–70. 10.1136/neurintsurg-2017-013308 28918385 PMC5969388

[26] Tanioka S Ishida F Yamamoto A Shimizu S Sakaida H Toyoda M. Machine learning classification of cerebral aneurysm rupture status with morphologic variables and hemodynamic parameters. Radiol Artif Intell. (2020) 2:e190077. 10.1148/ryai.2019190077 PMC8017392 33937812

[27] Kim HC Rhim JK Ahn JH Park JJ Moon JU Hong EP. Machine learning application for rupture risk assessment in small-sized intracranial aneurysm. J Clin Med. (2019) 8:683. 10.3390/jcm8050683 31096607 PMC6572384

[28] Aranda A Valencia A. Study on cerebral aneurysms: rupture risk prediction using geometrical parameters and wall shear stress with CFD and machine learning tools. Mach Learn Appl. (2018) 5:5401. 10.5121/mlaij.2018.5401

[29] Liu Q Jiang P Jiang Y Li S Ge H Jin H. Bifurcation configuration is an independent risk factor for aneurysm rupture irrespective of location. Front Neurol. (2019) 10:844. 10.3389/fneur.2019.00844 31447764 PMC6691088

[30] Dai X Huang L Qian Y Xia S Chong W Liu J. Deep learning for automated cerebral aneurysm detection on computed tomography images. Int J Comput Assist Radiol Surg. (2020) 15:715–23. 10.1007/s11548-020-02121-2 32056126

[31] Liu P Wang H Zheng S Zhang F Zhang X. Parkinson's disease diagnosis using neostriatum radiomic features based on T2-weighted magnetic resonance imaging. Front Neurol. (2020) 11:248. 10.3389/fneur.2020.00248 32322236 PMC7156586

[32] Chen Q Zhu D Liu J Zhang M Xu H Xiang Y. Clinical-radiomics nomogram for risk estimation of early hematoma expansion after acute intracerebral hemorrhage. Acad Radiol. (2020). 10.1016/j.acra.2020.02.021. [Epub ahead of print]. 32238303

[33] Liu Z Ji B Zhang Y Cui G Liu L Man S. Machine learning assisted MRI characterization for diagnosis of neonatal acute bilirubin encephalopathy. Front Neurol. (2019) 10:1018. 10.3389/fneur.2019.01018 31632332 PMC6779823

[34] Jang J Ngo LH Mancio J Kucukseymen S Rodriguez J Pierce P. Reproducibility of segmentation-based myocardial radiomic features with cardiac MRI. Radiol Cardiothoracic Imaging. (2020) 2:e190216. 10.1148/ryct.2020190216 32734275 PMC7377242

