# Supplementary material for: CT Angiography-Based Radiomics for Classification of Intracranial Aneurysm Rupture
Source: Front Neurol. 2021 Feb 22;12:619864. doi: 10.3389/fneur.2021.619864 (PMC7937935; doi:10.3389/fneur.2021.619864)
Supplement: Supplementary file 1 [file Table_1.DOCX]

Supplementary Material

# Imaging protocols

Standard brain CT angiography images were acquired on axial section with post-processing reconstruction on sagittal, coronal, maximum intensity projection (MIP) and 3-dimensional volume rendered (3D-VR) views as necessary.

Images from Wuhan Union Hospital were acquired using one of the following scanners utilizing the listed parameters:

1. Discovery CT750 HD (GE Healthcare, Chicago, IL, USA): slice thickness, 0.63 mm; tube voltage, 100 kVp; and effective tube current, 2-3 mAs.
2. SOMATOM Definition AS+ (Siemens Healthineers, Erlangen, Germany): slice thickness, 0.6 mm; tube voltage, 100 kVp; and effective tube current, 80–140 mAs.
3. Aquilion ONE (Toshiba, Tokyo, Japan): slice thickness, 0.5 mm; tube voltage, 100 kVp; and effective tube current, 150 mAs.

Images from Union West Hospital were acquired using Ingenuity CT scanner (Philips Healthcare, Best, The Netherlands) with slice thickness of 1.00mm; tube voltage of 120 kVp; and effective tube current of 200 mAs.
